# Exploring the Relationship between Frailty, Functional Status, Polypharmacy, and Quality of Life in Elderly and Middle-Aged Patients with Cardiovascular Diseases: A One-Year Follow-Up Study

**DOI:** 10.3390/ijerph19042286

**Published:** 2022-02-17

**Authors:** Elisabeta Ioana Hiriscau, Elena-Cristina Buzdugan, Ligia-Ancuta Hui, Constantin Bodolea

**Affiliations:** 1 Nursing Department, Faculty of Medicine, University of Medicine and Pharmacy “Iuliu Hatieganu”, 400349 Cluj-Napoca, Romania; 2 Anaesthesia and Intensive Care Unit, University Clinical Municipal Hospital, 400139 Cluj-Napoca, Romania; cbodolea@gmail.com; 3 Internal Medicine Department, Faculty of Medicine, University of Medicine and Pharmacy “Iuliu Hatieganu”, 400012 Cluj-Napoca, Romania; buzelena@yahoo.com; 4 Cardiology Unit, University Clinical Municipal Hospital, 400139 Cluj-Napoca, Romania; 5 Pharmaceutical Technology and Biopharmaceutics Department, Faculty of Pharmacy, University of Medicine and Pharmacy “Iuliu Hatieganu”, 400012 Cluj-Napoca, Romania; ligiahui@yahoo.com; 6 University Clinical Municipal Hospital, 400139 Cluj-Napoca, Romania; 7 Anaesthesia and Intensive Care Department, Faculty of Medicine, University of Medicine and Pharmacy “Iuliu Hatieganu”, 400012 Cluj-Napoca, Romania

**Keywords:** cardiovascular diseases, elderly, frailty, functional status, middle-aged patients, quality of life, polypharmacy

## Abstract

The association between frailty, disability in activities of daily living (ADL), polypharmacy, and quality of life (QoL) in middle-aged patients with cardiovascular disease (CVD) is little investigated. This study sought (a) to explore this association comparatively in elderly and middle-aged hospitalized patients with CVD and (b) to determine which domains of ADL and QoL might improve the frailty prediction. A one-year follow-up study including 90 elderly (≥65 years old) and 89 middle-aged patients (40–65 years old) was conducted. At baseline, frailty assessment was performed based on the Fried criteria; Barthel Index (BI) and Duke Activity Status Index (DASI) were used for ADL, and European Quality of Life-5 dimensions (EQ-5D) for QoL. At follow-up, data were collected via telephone. At baseline, 79 patients (51 elderly and 28 middle-aged) were frail. The CVD frail patients showed functional dependency and a poor QoL compared to the non-frail (*p* < 0.001) and within each subgroup at follow-up. Mobility was found to predict frailty in both elderly (OR = 2.34) (C.I. (1.03–5.29)) and middle-aged patients (OR = 2.58) (C.I. (1.15–5.78)). The ADL assessment and self-reported QoL may help to identify an aggravation or an advanced frailty condition in hospitalized elderly and middle-aged CVD patients.

## 1. Introduction

Frailty is a geriatric syndrome, defined as a reduced homeostatic reserve leading to increased vulnerability to stressors. It is also associated with a high risk for adverse health-related outcomes [1,2]. 

From a pathophysiological point of view, frailty syndrome is characterized by multiple dysfunctions at different levels (musculoskeletal, neuroendocrine, hematological, immune, cardiovascular) due to a state of chronic low-grade inflammation, expressed by an increase in inflammatory biomarkers like C-reactive protein (CRP), interleukin-6 (IL-6), and tumor necrosis factor alpha (TNF-α). Other inflammatory markers from peripheral blood cells, such as the lymphocyte count, neutrophils, platelets, and red distribution width (RDW), were also found to be associated with the severity of frailty [3,4,5,6,7].

### 1.1. Frailty Conceptualizations

Two emerging models are relevant to describe frailty: frailty phenotype and the model based on the accumulation of deficits. Within the phenotypic model, frailty is assessed through five dimensions, so called Fried criteria: (unintentional) loss of weight, exhaustion, low physical activity, weak grip strength, and slow walking speed [8]. An individual is considered frail if three or more physical criteria are present. Within the deficits accumulation model, frailty is assessed based on the frailty index (FI), an expression of measurement of the cumulative burden given by several symptoms, diseases, medical conditions, and functional decline [9,10]. According to this index, a higher score reflects an advanced frailty condition. 

### 1.2. Frailty, Functional Decline, and Quality of Life (QoL) in Cardiovascular Diseases (CVDs)

Due to the negative health outcomes associated with frailty, routine screening and studies have been applied and recommended to populations aged over 65 years [11,12,13,14,15], but there is limited data available regarding middle-aged populations (45–65 years old) or those younger than 45 years old [16,17]. 

In patients with CVDs, frailty has been proven to be a predictor of mortality, comorbidities, and disability [18]. Elderly CVD patients showed a risk of 2.7 to 4.1 for incident frailty and 1.5 for those who were not frail at baseline [19]. Frail older patients with chronic heart failure (HF) reported poor QoL because of recurrent hospitalizations, which increase their risk for additional deficits in functional performance, reduced mobility, falls, and polypharmacy [20,21]. In patients with acute decompensated HF, early functional decline after discharge is associated with an increase in activities of daily living (ADL) difficulty and a higher risk of re-admission or death over the next year [22,23]. 

Frailty is associated with increased risk of death, cardiovascular events, major bleeding, and stroke in patients aged over 65 years with acute coronary syndrome (ACS) [24,25]; in elderly patients with ACS, frailty was found to be the independent predictor of a worse QoL and was found to be associated with higher all-cause mortality [26,27,28]. In older patients with atrial fibrillation (AF), frailty was found to be an independent predictor of higher intensity of symptoms of arrhythmia and a worse Qol compared to non-frail individuals [29,30].

### 1.3. Arguments of the Study

The association between frailty and disability in ADL has been examined in multiple studies [31,32,33]. Three perspectives regarding the relationship between these concepts have been identified, with disability being (a) a negative health outcome of frailty, (b) a characteristic of frailty, and (c) a predictor of frailty [34]. The relationship between frailty and functional performance has been widely investigated in community-dwelling individuals with CVD aged 70 years old and over, but much less in middle-aged and younger individuals [27]. 

Although in many studies disability is considered a negative outcome of frailty, many clinicians consider the reverse approach to be equally important from a practical point of view [35,36]; therefore, we tested in this study whether functional status and QoL are associated, thus contributing to the improvement of the prediction of frailty status in patients with CVDs. 

We carried out a one-year follow-up study in a cohort of hospitalized elderly (aged 65 and over), middle-aged (45–65 years age old), and younger CVD patients, with the aim of exploring the relationship between frailty, functional performance, polypharmacy, and QoL. The second objective of the study was to identify the domains of ADL and QoL that predict frailty in elderly and in middle-aged individuals with CVDs. 

## 2. Materials and Methods

### 2.1. Study Sample

The sample was selected from the Frail.ro mother study (initial study), an institutional project developed by the University Clinical Municipal Hospital, Cluj-Napoca, Romania, which ran 2016–2019, aiming to identify the prevalence of the frailty syndrome in hospitalized patients in different clinical units. The inclusion criteria were the following:

- patients previously diagnosed with heart symptoms or CVD;
- patients admitted to the cardiology unit between July and December 2017;
- patients who agreed to participate and be assessed for frailty and signed the informed consent.

The exclusion criteria were dementia condition and delirium, chronic inflammatory diseases, and individuals who refused the comprehensive frailty assessment. 

In performing the present study, we extracted the baseline data of the CVD patients related to frailty condition, functional status, QoL, and medical treatment prescribed at discharge from the initial study. At follow-up we collected the data related to functional status and QoL by telephone. 

The study was conducted in accordance with the ethical principles stated in the Declaration of Helsinki in conducting medical research. Each patient enrolled in the study gave written consent after they had been informed about the purpose of the study, the procedures involved, and confidentiality and its limitations regarding the provided data. The study protocol was approved by the Local Ethics Committee of the University Clinical Municipal Hospital, Cluj-Napoca, Romania (reference Protocol nr. 5/2017, the approval of the study 20 February 2017). 

### 2.2. Variables

At baseline we collected demographic data, environmental characteristics, data related to lifestyle, self-perceived health, income, falls, and polypharmacy (a number equal to or more than five medications taken daily was considered a positive criterion). We used the stratification method for age, with the following ten age categories: <45, 45–49, 50–55, 55–60, and 60–<65 for the middle-aged group, and ≥65–69, 70–74, 75–79, 80–84, and >85 years for the elderly group. Alcohol consumption was quantified in units per day and per week [37]. For body mass index (BMI = weight/height^2^), we used the following cut-off values: <25 kg/m^2^ for normal weight, ≥25 kg/m^2^ for overweight, and ≥30 kg/m^2^ for obese patients. Other variables included in the analysis were those related to cardiac diseases (categorical variables), medications (categorical variables), and Charlson Comorbidity Index (CCI).

Frailty was measured using the Fried criteria, which operationalize physical frailty through the measurement of five characteristics:

1. Unintentional weight loss (≥5% of body weight in the last year).
2. Self-reported exhaustion was considered positive if the patient answered yes to either of the following questions extracted from the Center for Epidemiologic Studies Depression Scale [38]: “I felt that everything I did was an effort” or “I could not get going”. The respondents who answered positively were asked thereafter to evaluate how often they felt that way during the last week. A score of 0 = none of the time or rarely (<1 day) or 1 = some or a little of the time (1–2 days) was considered negative; a score of 2 = a moderate amount of the time (3–4 days) or 3 = most of the time defined the criterion as positive.
3. Weakness, measured as the mean grip strength of the dominant hand three times with a Jamar hydraulic dynamometer [39]; we used the cut-off values of 30 kg for men and 20 kg for women.
4. Slowed motor performance, measured by performing the 4–6 m speed test, adjusted for sex and height, according to the standards of the Short Physical Performance Battery [40].
5. Low energy expenditure (Kcal spent per week) as result of physical activity (PA) reported per 24 h and per week. PA was quantified using the International Physical Activity Questionnaire (IPAQ) short form, translated into the Romanian language. The procedure of the PA measurement is described in detail elsewhere [7].

Functional status was measured using the Barthel Index (BI), which evaluates the ability of the patients over 65 years old to perform ADL. Scores range from 0 to 100, in steps of 5, with a higher ADL score indicating better functional status [41,42]. Functional autonomy was classified as follows: totally dependency (0–20 points), severe (21–60 points), moderate (61–90 points), slight (91–99 points), and independence (100 points). 

The Duke Activity Status Index (DASI) was used to measure the functional capacity of CVD patients aged less than 65 years. The highest score is 58.2. A DASI score greater than 34 but less than 58.2 represents a good functional capacity. A DASI score of 34 or less means that the patient is at risk of myocardial injury, myocardial infarction, moderate-to-severe complications, and new disabilities [43,44]. 

The number of prescribed drugs at discharge, including β-blockers, angiotensin converting enzyme inhibitors (ACE), diuretics, nitrates, calcium channel blockers, statins, anticoagulants, antidiabetic drugs, and antiarrhythmic drugs, were measured by their presence or absence. 

QoL was assessed using the European Quality of Life-5 dimensions (EQ-5D) instrument. The version EQ-5D-5L contains 5 dimensions, with 5 levels of response options, which reflect no problems (level 1), slight problems (level 2), moderate problems (level 3), severe problems (level 4), and extreme problems (level 5). The participant is asked to indicate his/her health state by ticking the box next to the most appropriate statement corresponding to each evaluated domain [45,46]. 

### 2.3. Follow-Up

The one-year follow-up was performed by telephone. A form, to assist in comparing the results from the baseline and follow-up evaluation, was used during the telephone interview. This form contained the code of the patient (assigned at the baseline assessment), the patient’s telephone number, and the results of the baseline assessment regarding functional status and QoL. During the interview, each participant was asked to respond to the items according to the Barthel Index or DASI, and QoL. 

### 2.4. Statistical Analysis

For statistical analysis we considered 2 groups: the non-frail group, which included patients fulfilling none (robust), 1 or 2 frailty criteria (pre-frail), and the frail group, which included patients with 3 or more criteria. Each group was divided into two subgroups: elderly patients, non-frail and frail; and middle-aged patients, non-frail and frail. 

Continuous variables were expressed as the mean and standard deviation, and categorical variables as the absolute value with their percentage. We used the chi-squared test for comparison of the categorical variables. The linear correlations between continuous variables were evaluated using Pearson and Spearman’s rank test, respectively. Independent sample t-tests and Mann-Whitney tests were used at baseline for the differences between non-frail and frail elderly patients and non-frail and frail middle-aged patients. At follow-up, for identifying the differences within each subgroup, we used the Wilcoxon non-parametric test. 

Predictive models, using the dichotomized frailty classification, were developed as follows: all variables showing correlation with frailty (r = >0.3) were entered one by one in the linear regression for checking the multicollinearity and regression assumptions; the selected variables were entered in the binary logistic regression for testing the predictive models for each group. Regression models included adjustment for age and gender. 

Statistical significance was set at a *p*-value of less than 0.05. Data analyses were carried out using IBM SPSS Statistics for Windows, version 20.0 (IBM Corp., Armonk, NY, USA).

## 3. Results

### 3.1. Baseline Characteristics

The sample included 179 patients at baseline, 90 patients aged 65 and over (elderly group), and 89 patients aged 39–65 years (middle-aged group). Out of 179 patients, 78 (43.6%) were men, and 101 (56.4%) were women. At baseline, 79 (44.1%) patients from the sample were identified with frailty. The frail group included 51 (64.6%) elderly patients and 28 (35.4%) middle-aged patients. A total of 100 (55.9%) robust and pre-frail patients were grouped together in the non-frail group, which included 39 (39%) elderly and 61 (61%) middle-aged patients. The sample baseline characteristics corresponding to all four subgroups (the non-frail group included the non-frail elderly and non-frail middle-aged subgroups; the frail group included the frail elderly and frail middle-aged subgroups) are illustrated in Table 1. 

At baseline, the non-frail elderly CVD patients were less educated, more likely to live single or without a partner, smoked less, had more comorbidities and were more likely to take more than five medications daily, and had a poorer QoL in comparison with non-frail middle-aged individuals. Statistically significant differences between non-frail subgroups, with higher levels in elderly patients, were found with regard to ischemic heart disease, hypertension, cardiac valvulopathies, pacemakers, congestive heart failure, NYHA classification in the patients with heart failure, and the use of diuretics and anticoagulants. 

The differences in means between frail subgroups were related to gender, education level, marital status, smoking, falls, comorbidities, cardiac valvulopathies, and the use of diuretics and anticoagulants. 

### 3.2. Comparisons between Groups at Baseline 

The differences in means between non-frail and frail elderly CVD patients, and non-frail and frail middle-aged patients, are presented in Table 2. BI scores characterize the elderly patients and DASI scores describe the middle-aged patients included in the research sample. 

At baseline, statistically significant differences between CVD elderly subgroups, with higher levels in the frail patients, were found with regard to age, living alone or without a partner, falls, lower education level, poor self-perceived health, lower income, cardiac valvulopathies, and the use of diuretics and nitrates. The differences in means between CVD middle-aged subgroups were related to lower income, more comorbidities, ischemic heart disease, congestive heart failure, and the use of diuretics, with higher levels in frail patients.

The statistically significant differences between CVD elderly subgroups concerning QoL were found with regard to mobility, self-care, and usual activities, with higher scores in the frail subgroup; between middle-aged subgroups, the differences were found to be related to mobility, self-care, usual activities, and anxiety/depression, with higher scores characterizing the frail subgroup. 

Statistically significant differences between elderly subgroups regarding functional status were related to feeding, bathing, grooming, dressing, toilet use, transfers, mobility, and stairs, with lower levels in frail patients; between middle-aged subgroups, the differences were found with regard to all DASI domains, with lower scores describing the frail subgroup. Related to functional autonomy, both frail elderly and frail middle-aged patients proved to be less autonomous than non-frail individuals.

### 3.3. Survival at One-Year Follow-Up

**Figure 1:**
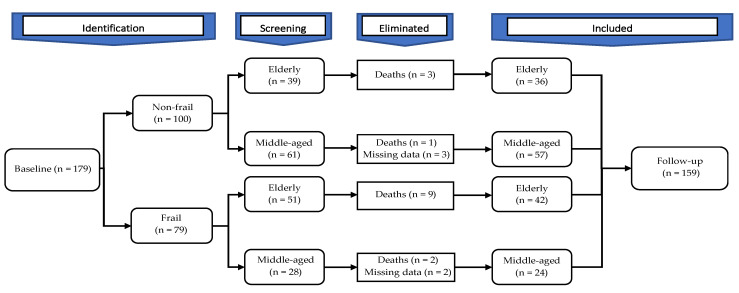
The chart with the sample evolution at one-year follow-up.

At follow-up, 15 patients out of the total sample, 12 elderly (3 non-frail and 9 frail), and 3 middle-aged patients (1 non-frail and 2 frail), were lost from the study (Figure 1). 

**Figure 2:**
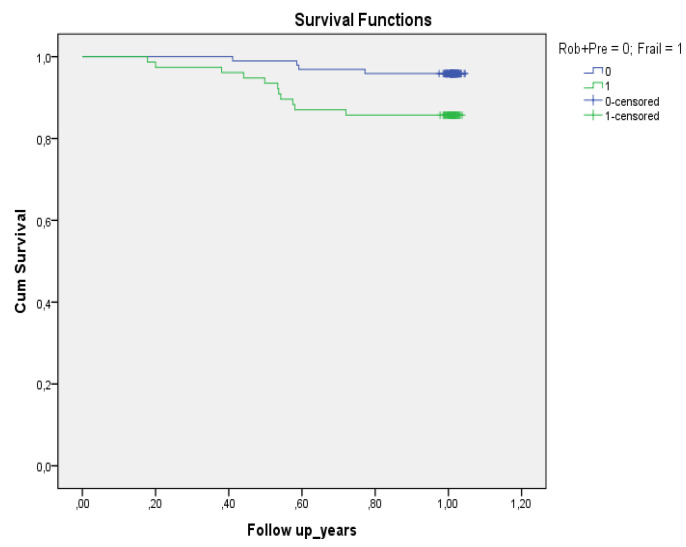
Survival at follow-up (12 months).

The mortality rate after one year from baseline was 8.4%: 13.3% in the elderly group and 3.4% in the middle-aged group. Five individuals had missing data from middle-aged group (3 non-frail and 2 frail) at follow-up (3.1%). The survival functions (Kaplan–Meier test) are presented in Figure 2. The test of equality of survival distribution (Log Rank) showed a statistically significant difference (*p* = 0.016) between different levels of non-frail and frail patients with CVD. 

### 3.4. Intra-Group Comparisons at Follow-Up

The follow-up data presented in the Table 3 and Table 4 show the differences related to functional status and QoL within each subgroup of patients.

The non-frail elderly CVD patients showed lower control in bladder functioning compared to baseline, whereas the frail elderly were less autonomous in performing activities related to feeding, bathing, bladder control, and mobility on level surfaces. The non-frail elderly CVD patients showed less satisfaction in relation to self-care and usual activities compared to baseline; the frail elderly CVD subgroup reported less satisfaction in performing self-care, more pain/discomfort, and higher levels of anxiety/depression. 

The non-frail middle-aged CVD patients showed less capacity in doing heavy work around the house and reported reduced satisfaction in all QoL domains; the frail middle-aged CVD patients showed diminished capacity in performing light work around the house and less satisfaction related to mobility, self-care, and usual activities, and higher levels of pain, compared to baseline assessment. 

Within the logistic regression analysis, the covariates for elderly patients CVD were BI 4 (dressing), BI 7 (toilet use), BI 8 (transfers), BI 9 (mobility on level surfaces), BI 10 (stairs), and mobility, self-care, and usual activities as QoL domains. The covariates for the middle-aged CVD group were DASI 4 (climbing stairs), DASI 6 (doing light work around the house), DASI 7 (doing moderate work around the house), DASI 8 (doing heavy work around the house), DASI 9 (doing garden work), DASI 10 (sexual relations), and mobility as the QoL domain. The models were adjusted for age and gender. The data are shown in Table 5 and Table 6.

## 4. Discussion

In this article we present novel study findings. The data reported in meta-analyses regarding the relationship between frailty and disability showed a higher risk for incident and worsening ADL or for combined disability in frail individuals (OR = 4.44) compared with the non-frail [47,48]. Our data show a high risk for incident disability in CVD frail elderly of 4.4-fold (OR = 4.4) (C.I. (1.8–10.7)), and of 12.9-fold (OR = 12.93) (C.I. (4.2–39.8)) in CVD frail middle-aged compared to the non-frail. 

The relation between frailty, falls, and functional autonomy has been investigated in various studies [8,49]. In our study we found negative correlations between falls, transfers (r = −0.37, *p* < 0.001), and mobility on level surfaces (r = −0.32, *p* = 0.002) in the elderly CVD group, with a higher number of falls being associated with a lower score in ADL (r = −0.45, *p* < 0.001). A negative correlation between falls and age was found, with the more vulnerable individuals being those aged 70–74 (Supplementary material/Table S1). No association between falls and functional performance (BI total score) characterized the non-frail elderly subgroup; in contrast, falls and functional performance (r = −0.47, *p* < 0.001) were negatively correlated in frail elderly patients. Although a positive correlation between falls and frailty (r = 0.29, *p* = 0.006) was identified in elderly hospitalized CVD patients, with frail patients reporting more falls than the non-frail, the falls were not found relevant in predicting frailty in the elderly group (Supplementary material/Table S1). 

With regard to the relationship between functional status and QoL in non-frail elderly CVD patients at baseline, a negative correlation between BI score and self-care was found (Supplementary material/Table S2). Between cardiac valvulopathies, BI, and QoL total scores, no significant correlations were found, but BI 6 (bladder control) and BI 10 (ascending and descending stairs) were negatively correlated with cardiac valvulopathies (Supplementary material/Table S3). In frail elderly CVD patients, no significant correlations were found with regard to cardiac valvulopathies, BI, and QoL total scores. At follow-up, impaired functional status was reported when the non-frail patients had worse mobility, self-care, and usual activities, and higher levels of anxiety/depression; in the frail elderly, lower scores of functional dependency were negatively correlated with worsening of all QoL domains (Supplementary material/Tables S2 and S4).

Impaired mobility was found to predict frailty in the elderly CVD group (OR = 2.34) (C.I. (1.03–5.29)), with the elderly patients with affected mobility 2.34-fold more likely to become frail. Mobility limitations were associated with increased falls, disability, hospitalization, mortality risk, and a decreased QoL in older adults [50,51]. Still, there is a potential bias related to mobility and cardiac disease. Walking speed is a marker of mobility and an independent predictor of disability. It is known that ischemic heart disease, AF, HF, and stroke are strongly associated with an increase of mobility limitation. This association might be explained by decreased physiological reserve, which is characteristic for frailty condition, or by low oxygenation or atherosclerotic changes, which occur in CVDs [52,53].

Regarding age, the elderly CVD patients aged 75–79 years old were 7.65-fold more vulnerable to the risk of becoming frail (C.I. (1.15–50.96)) according to our data. This age group was localized as being more exposed in developing frailty, especially in acute decompensated heart failure (ADHF), which is also associated with a poor QoL [23]. In a cohort of older patients with a mean age of 72 years old with ADHF, frailty was associated with worse physical function, comorbidity, depression, and a reduced general QoL, including mobility, usual activities, and higher pain/discomfort levels [54].

Hanlon et al. reported a significant association between frailty and comorbidities, and mortality in middle-aged individuals [55]. Our data show that the middle-aged frail CVD patients are characterized by more comorbidities than the non-frails, but a significant correlation between these parameters was not found.

With regard to functional capacity, our data reveal a negative correlation between falls and DASI total score (r = −0.32, *p* = 0.003), with a high number of falls being associated with a low DASI score in middle-aged CVD patients. In the non-frail middle-aged subgroup, a negative correlation between falls and DASI total score (r = −0.42, *p* = 0.001) was found, but there was no correlation in the frail subgroup, which might be explained by the fact that other risk factors, such as severity of the disease or acute episodes/exacerbations or associated comorbidities, are more relevant for the frailty condition (Supplementary material/Table S5). Like the elderly group, falls were not found to predict frailty in middle-aged CVD patients. At follow-up, both CVD middle-aged subgroups showed a diminished functional capacity in comparison to the baseline: the non-frail patients in doing heavy work around the house, and the frail patients in performing light work around the house.

Negative correlations between DASI total score and QoL domains were found in the middle-aged group, with a low functional capacity being associated with higher difficulties in mobility, less satisfaction in performing self-care, usual activities, and with high levels of anxiety/pain. Fan et al. reported in their study that impaired functional status (lower DASI score) was associated with a worse health-related quality of life [56]. In our study no significant correlations between DASI total score and QoL domains were found in non-frail middle-aged patients at baseline. Significant correlations were found between ischemic heart disease and DASI total score, DASI 5 (running a short distance), DASI 7 (doing moderate work around the house), DASI 11 (participating in moderate recreational activities), and DASI 12 (participating in strenuous sports). DASI score and QoL domains measured at follow-up showed that non-frail middle-aged patients reported a depreciated functional capacity (low DASI score) when they had worse mobility, self-care, and usual activities, and higher levels of pain (Supplementary material /Tables S6 and S7). In frail middle-aged CVD patients, a positive correlation was found between heart failure and DASI 3 (walking on level ground). The DASI scores were negatively correlated with all QoL domains, with the frail patients reporting impaired functional status when they had all QoL domains aggravated (Supplementary material/Tables S8 and S9).

According to our data, the patients aged 60–65 years old were 64.18-fold more exposed to the risk of becoming frail of (C.I. (1.17–3532.14)). From a chronological perspective, compared to younger individuals, this age group is the most vulnerable to develop frailty, with an advanced age being a significant risk factor for frailty. In selected populations with specific diseases or conditions, such as patients with cancer, end-stage renal disease, and heart failure, frailty has a high prevalence, regardless of age [12,57]. Although the trajectory of decline with ageing varies widely between individuals, the occurrence of functional disability after a myocardial infarction or a stroke in a middle-aged individual may be closely linked to the development of frailty.

In the middle-aged group, the capacity in doing heavy work around the house (*p* = 0.002) and mobility (*p* = 0.022) were found to better predict frailty. Related to mobility limitations, the middle-aged CVD patients reporting more difficulties in mobility were more likely to become frail (OR = 2.58) (C.I. (1.15–5.78)). A decreased capacity in doing heavy work around the house (e.g., scrubbing floors, lifting or moving heavy furniture) might be seen as an alarm signal for the occurrence of deficits that are suggestive for pre-frailty in middle-aged patients.

A few limitations regarding the study warrant consideration. Our results must be interpreted with caution. First, patients were recruited from a single medical center. Second, given the variability between patients due to the cardiac illness and outcomes, the sample size is modest. The sample included 179 participants, with different cardiac pathology, divided in two groups: elderly and middle-aged. Each group was split into frail and non-frail subgroups. Robust and pre-frail patients were pooled together due to the low number of patients classified as pre-frail, which precluded a comparison of all three frailty categories (frail, pre-frail, and robust). Another limit of the study is reflected by the fact that the non-frail group also included patients with one or two frailty criteria; this aspect could have influenced the differences between the non-frail and frail subgroups related to some parameters that were at the limit of being relevant to frailty condition. Another bias might also be related to the accuracy of follow-up data collection; the data is rather subjective due to the telephone interview, being exclusively based on the patients’ self-reports.

## 5. Conclusions

Frailty is associated with a worse quality of life and poor prognosis in patients with CVD. A difficulty in performing activities of daily living associated with a self-reported altered quality of life, for young patients with CVD, might be an element to include in the global evaluation of appropriate frailty parameters. Future research should include methods to select and stratify participants with frailty, especially the middle-aged or younger CVD population at risk, as well as the corresponding validated instruments in performing frailty assessment in cardiac patients. Clinical data on cardiovascular function need to be assessed prospectively to evaluate the effect of the severity of certain cardiovascular diseases on frailty.

## Figures and Tables

**Table 1:** Baseline characteristics of the sample included in the analysis. Quantitative variables are expressed as mean (M) and standard deviation (SD), and qualitative variables as a percentage.

Baseline Characteristics							
Variables		Non-Frail Group (*n* = 100)			Frail Group (*n* = 79)		
		Elderly (*n* = 39)	Middle-Aged (*n* = 61)	*p*-Value	Elderly (*n* = 51)	Middle-Aged (*n* = 28)	*p*-Value
		Mean (SD) or *n* (%)			Mean (SD) or *n* (%)		
Age (years)		72.3 (5.7)	54.1 (8.5)		77.9 (6.0)	55.4 (9.0)	
Gender	Males	16 (41)	34 (56)	0.154	12 (24)	16 (57)	**0.002**
	Females	23 (59)	27 (44)		39 (76)	12 (43)	
Environment origin	Urban	16 (41)	36 (59)	0.081	21 (41)	14 (50)	0.088
	Rural	23 (59)	25 (41)		30 (59)	14 (50)	
Education level	Elementary	18 (46)	11 (18)	**0.021**	42 (82)	4 (14)	***p* < 0.001**
	Secondary	14 (36)	36 (59)		6 (12)	20 (71)	
	Bachelor	7 (18)	14 (23)		3 (6)	4 (14)	
Marital/Civil status	Married	23 (59)	53 (87)	**0.001**	18 (35)	21 (75)	**0.001**
	Single	16 (41)	8 (13)		33 (65)	7 (25)	
Smoking	Non-Smoker	38 (97)	52 (85)	**0.048**	50 (98)	22 (79)	**0.003**
	Active Smoker	1 (3)	9 (15)		1 (2)	6 (21)	
Alcohol consumption ^a^	<7(14) u/week	33 (85)	58 (95)	0.076	58 (95)	27 (96)	0.323
	>7(14) u/week	6 (15)	3 (5)		3 (5)	1 (4)	
Self-perceived health	Poor	32 (82)	49 (80)	0.832	51 (100)	26 (93)	0.054
	Good	7 (18)	12 (20)		0	2 (7)	
Sleeping	Poor	29 (74)	49 (80)	0.487	41 (80)	24 (86)	0.559
	Good	10 (26)	12 (20)		10 (20)	4 (14)	
Falls (within the last year)	No falls	27 (69)	44 (72)	0.758	19 (37)	19 (68)	**0.009**
	Falls	12 (31)	17 (28)		32 (63)	9 (32)	
Income	<MGWpE ^b^	12 (31)	18 (30)	0.895	31 (61)	17 (60)	0.995
	>MGWpE	27 (69)	43 (70)		20 (39)	11 (40)	
Polypharmacy (>5 medications taken daily)		18 (46)	15 (25)	**0.025**	31 (61)	11 (39)	0.071
BMI ^c^	BMI < 25	13 (33)	17 (28)	0.601	17 (33)	7 (25)	0.715
	BMI > 25–<30	22 (57)	37 (61)		27 (53)	18 (64)	
	BMI > 30	4 (10)	7 (11)		7 (14)	3 (11)	
Charlson Comorbidity Index		6.1 (2.1)	4.2 (2.1)	***p* < 0.001**	6.9 (2.3)	5.4 (2.9)	**0.014**
EQOL-5D-5L ^d^ (total score)		7.4 (2.1)	7.2 (2)	**0.012**	11.1 (3.8)	10.6 (4.5)	0.634
Cardiac diseases		Elderly non-frail (*n* = 39)	Middle-aged non-frail (*n* = 61)		Elderlyfrail (*n* = 51)	Middle-aged frail (*n* = 28)	
Ischemic heart disease		26 (67)	26 (43)	**0.019**	39 (77)	20 (71)	0.627
Arterial hypertension		34 (87)	42 (69)	**0.037**	46 (90)	21 (75)	0.073
Cardiac valvulophathies		25 (64)	26 (43)	**0.036**	43 (84)	17 (61)	**0.019**
Arrhythmias		19 (49)	24 (39)	0.361	35 (69)	15 (54)	0.189
Conduction disorders		5 (13)	3 (5)	0.159	2 (4)	3 (11)	0.241
Pacemaker		5 (13)	0 (0)	**0.004**	4 (8)	1 (4)	0.462
Congestive heart failure		20 (52)	16 (26)	**0.011**	36 (71)	15 (53)	0.134
NYHA ^e^ Classification	I	0 (0)	2 (3)	**0.001**	0 (0)	1 (4)	0.147
	II	10 (26)	13 (21)		13 (26)	6 (21)	
	III	10 (26)	1 (2)		22 (43)	6 (21)	
	IV	0 (0)	0 (0)		1 (2)	2 (7)	
Medications prescribed at discharge		Elderly non-frail (*n* = 39)	Middle-aged non-frail (*n* = 61)		Elderly frail (*n* = 51)	Middle-aged frail (*n* = 28)	
β-blockers		29 (74)	34 (58)	0.061	33 (65)	18 (64)	0.971
ACE ^f^ inhibitors		21 (54)	28 (46)	0.444	35 (69)	14 (50)	0.105
Diuretics		26 (67)	23 (38)	**0.004**	45 (88)	17 (61)	**0.004**
Nitrates		5 (13)	11 (18)	0.493	18 (35)	7 (25)	0.353
Anticoagulants		33 (85)	28 (46)	***p* < 0.001**	47 (92)	16 (57)	***p* < 0.001**
Calcium channel blockers		14 (35)	15 (25)	0.228	15 (29)	10 (36)	0.570
Statins		16 (41)	20 (33)	0.408	13 (26)	10 (38)	0.345
Antidiabetic drugs		5 (13)	3 (5)	0.159	6 (12)	6 (21)	0.258
Antiarrhythmic drugs		5 (13)	5 (8)	0.457	9 (18)	2 (7)	0.202

Notes: Variables in bold are significant at *p* < 0.05. ^a^ Alcohol consumption (1 unit = 250 mL beer or 75 mL wine or 25 mL brandy); ^b^ Minimum gross wage per economy; ^c^ Body Mass Index (kg/m^2^); ^d^ European Quality of Life -5 dimensions-5 levels; ^e^ New York Heart Association; ^f^ Angiotensin-converting enzyme.

**Table 2:** Mean differences between elderly patients (non-frail and frail) and middle-aged patients (non-frail and frail) at baseline. Quantitative variables are expressed as mean (M) and standard deviation (SD), and qualitative variables as a percentage.

		Elderly Group (*n* = 90)			Middle-Aged Group (*n* = 89)		
Variables		Elderly Non-Frail (*n* = 39)	Elderly Frail (*n* = 51)	*p*-Value	Middle-Aged Non-Frail (*n* = 61)	Middle-Aged Frail (*n* = 28)	*p*-Value
		Mean (SD) or *n* (%)			Mean (SD) or *n* (%)		
Age		72.3 (5.7)	77.9 (6)	***p* < 0.001**	54.1 (8.5)	55.3 (9)	0.532
Gender	Males	16 (41)	12 (24)	0.084	34 (56)	16 (57)	0.903
	Females	23 (59)	39 (76)		24 (44)	12 (43)	
Environment origin	Urban	16 (41)	21 (41)	0.989	36 (59)	14 (50)	0.432
	Rural	23 (59)	30 (59)		25 (41)	14 (50)	
Education level	Elementary	18 (46)	42 (82)	**0.001**	11 (18)	4 (14)	0.727
	Secondary	14 (36)	6 (12)		36 (59)	20 (71)	
	Bachelor	7 (18)	3 (6)		14 (23)	4 (14)	
Marital/civil status	Married	23 (59)	18 (35)	**0.025**	53 (87)	21 (75)	0.213
	Single	16 (41)	33 (65)		8 (13)	7 (25)	
Smoking	Non-Smoker	38 (97)	50 (98)	0.850	52 (85)	22 (79)	0.440
	Active Smoker	1 (3)	1 (2)		9 (15)	6 (21)	
Alcohol consumption	<7(14) u/week	33 (85)	58 (95)	0.429	58 (95)	27 (96)	0.779
	>7(14) u/week	6 (15)	3 (5)		3 (5)	1 (4)	
Self-perceived health	Poor	32 (82)	51 (100)	**0.001**	49 (80)	26 (93)	0.135
	Good	7 (18)	0		12 (20)	2 (7)	
Sleeping	Poor	29 (74)	41 (80)	0.501	49 (80)	24 (86)	0.544
	Good	10 (26)	10 (20)		12 (20)	4 (14)	
Falls (within the last year)	No falls	27 (69)	19 (37)	**0.002**	44 (72)	19 (68)	0.685
	Falls	12 (31)	32 (63)		17 (28)	9 (32)	
Income	<MGWpE	12 (31)	31 (61)	**0.004**	18 (30)	17 (60)	**0.005**
	>MGWpE	27 (69)	20 (39)		43 (70)	11 (40)	
Polypharmacy (>5 medications taken daily)		18 (46)	31 (61)	0.171	15 (25)	11 (39)	0.160
BMI ^a^	BMI < 25	13 (33)	17 (33)	0.802	17 (28)	7 (25)	0.789
	BMI >25–<30	22 (57)	27 (53)		37 (61)	18 (64)	
	BMI > 30	4 (10)	7 (14)		7 (11)	3 (11)	
Charlson Comorbidity Index		6.1 (2.1)	6.9 (2.3)	0.115	4.2 (2.1)	5.4 (2.9)	**0.032**
Cardiac diseases		Elderly non-frail (*n* = 39)	Elderly frail (*n* = 51)	*p*-Value	Middle-aged non-frail (*n* = 61)	Middle-aged frail (*n* = 28)	*p*-Value
		Mean (SD) or *n* (%)			Mean (SD) or *n* (%)		
Ischemic heart disease		26 (67)	39 (77)	0.316	26 (43)	20 (70)	**0.010**
Arterial hypertension		34 (87)	46 (90)	0.656	42 (69)	21 (75)	0.551
Cardiac valvulophathies		25 (64)	43 (84)	**0.034**	26 (43)	17 (61)	0.117
Arrhythmias		19 (49)	35 (69)	0.06	24 (39)	15 (54)	0.221
Conduction disorders		5 (13)	2(4)	0.149	3 (5)	3 (11)	0.383
Pacemaker		5 (13)	4 (8)	0.455	0 (0)	1 (4)	0.326
Congestive heart failure		20 (52)	36 (71)	0.066	16 (26)	15 (54)	**0.018**
Medications prescribed at discharge		Elderly non-frail (*n* = 39)	Elderly frail (*n* = 51)	*p*-Value	Middle-aged non-frail (*n* = 61)	Middle-aged frail (*n*= 28)	*p*-Value
		Mean (SD) or % (*n*)			Mean (SD) or % (*n*)		
β-blockers		29 (74)	33 (65)	0.327	34 (56)	18 (64)	0.453
ACE ^b^ inhibitors		21 (54)	35 (69)	0.160	28 (46)	14 (50)	0.723
Diuretics		26 (67)	45 (88)	**0.018**	23 (38)	17 (61)	**0.043**
Nitrates		5 (13)	18 (35)	**0.011**	11 (18)	7 (25)	0.453
Anticoagulants		33 (85)	47 (92)	0.284	28 (46)	16 (57)	0.330
Calcium channel blockers		14 (36)	15 (29)	0.520	15 (25)	10 (36)	0.283
Statins		16 (41)	13 (26)	0.127	20 (33)	10 (38)	0.789
Antidiabetic drugs		5 (13)	6 (12)	0.881	3 (5)	6 (21)	0.060
Antiarrhythmic drugs		5 (13)	9 (18)	0.537	5 (8)	2 (7)	0.866
EQ-5D-5L ^c^ total score		7.4 (2.1)	11.1 (3.8)	***p* < 0.001**	7.2 (2)	10.6 (4.5)	***p* < 0.001**
Mobility		1.6 (0.9)	2.8 (1)	***p* < 0.001**	1.3 (0.6)	2.3 (1.2)	***p* < 0.001**
Self-care		1.1 (0.3)	1.9 (1.1)	***p* < 0.001**	1 (0.3)	1.8 (1.1)	**0.002**
Usual activities		1.4 (0.5)	2.5 (1.1)	***p* < 0.001**	1.3 (0.6)	2.1 (1.2)	**0.002**
Pain/Discomfort		1.8 (0.9)	2.1 (0.9)	0.123	1.9 (0.9)	2.3 (0.9)	0.099
Anxiety/Depression		1.5 (0.9)	1.8 (1)	0.177	1.6 (0.8)	2.2 (1)	**0.003**
BI ^d^ total score		91.4 (15.6)	79.9 (18.5)	**0.002**			
BI 1 (feeding)		10 (0)	8.7 (2.6)	**0.001**			
BI 2 (bathing)		4.7 (1.1)	3.8 (2.2)	**0.005**			
BI 3 (grooming)		4.7 (1.1)	3.9 (2)	**0.018**			
BI 4 (dressing)		9.6 (1.4)	8.3 (2.4)	**0.002**			
BI 5 (bowel control)		9.2 (1.8)	9 (2)	0.608			
BI 6 (bladder control)		9.1 (1.9)	8.5 (2.3)	0.204			
BI 7 (toilet use)		9.7 (1.1)	8.3 (2.6)	**0.001**			
BI 8 (transfers)		14.3 (1.7)	12.4 (3.7)	**0.001**			
BI 9 (mobility on level surfaces)		14.2 (1.8)	12.1 (3.8)	**0.001**			
BI 10 (stairs)		7.8 (3)	5.4 (3.6)	**0.001**			
Functional autonomy BI	Dependency			***p* < 0.001**			
	Severe	1 (3)	10 (20)				
	Moderate	12 (30)	25 (49)				
	Slight	8 (21)	11 (22)				
	Independence	18 (46)	5 (10)				
DASI ^e^ total score					42.9 (13.4)	20.7 (3.3)	***p* < 0.001**
DASI 1 (self-care)					2.7 (0.4)	2 (1.2)	**0.007**
DASI 2 (walking indoors)					1.8 (0)	1.3 (0.8)	**0.006**
DASI 3 (walking on level ground)					2.8 (0)	2 (1.3)	**0.003**
DASI 4 (climbing stairs)					5.1 (1.4)	2.9 (2.8)	***p* < 0.001**
DASI 5 (running a short distance)					4.6 (4)	1.1 (2.9)	***p* < 0.001**
DASI 6 (doing light work around the house)					2.7 (0.3)	2.1 (1.1)	**0.02**
DASI 7 (doing moderate work around the house)					3.2 (0.9)	2 (1.8)	**0.001**
DASI 8 (doing heavy work around the house)					6.6 (3)	2 (3.5)	***p* < 0.001**
DASI 9 (doing garden work)					3.4 (2)	1.9 (2.3)	**0.005**
DASI 10 (sexual relations)					3.7 (2.4)	1.3 (2.3)	***p* < 0.001**
DASI 11 (participating in moderate recreational activities)					3.5 (3)	0.9 (2.1)	***p* < 0.001**
DASI 12 (participating in strenuous sports)					2.6 (3.6)	0.5 (2)	***p* < 0.001**
Functional		Autonomy			15 (25)	2 (7)	***p* < 0.001**
autonomy		Good			30 (49)	3 (11)	
**DASI**		At risk			16 (26)	23 (82)	

Notes: Variables in bold are significant at *p* < 0.05. ^a^ Body Mass Index; ^b^ Angiotensin-converting enzyme; ^c^ European Quality of Life-5 dimensions-5 levels; ^d^ Barthel Index; ^e^ Duke Activity Status Index.

**Table 3:** Mean differences regarding functional status and QoL within elderly group at follow-up. Quantitative variables are expressed as mean (M) and standard deviation (SD), and qualitative variables as a percentage.

		Elderly Group				
	Baseline Non-Frail (*n* = 39)	Follow-Up Non-Frail (*n* = 36)	*p*-Value	Baseline Frail (*n* = 51)	Follow-Up Frail (*n* = 42)	*p*-Value
	Mean (SD) or *n* (%)			Mean (SD) or *n* (%)		
BI ^a^ total score	91.3 (16.1)	90.1 (10.9)	0.717	80.7 (18.2)	74.5 (21.4)	**0.024**
BI 1 (feeding)	10 (0)	9.7 (1.2)	0.16	8.9 (2.6)	7.5 (3.5)	**0.009**
BI 2 (bathing)	4.7 (1.2)	4.7 (1.7)	1.0	3.9 (2.1)	3 (2.5)	**0.01**
BI 3 (grooming)	4.9 (0.8)	5 (0)	0.324	4.2 (1.9)	4.2 (2.2)	1.0
BI 4 (dressing)	9.6 (1.4)	9.2 (1.9)	0.083	8.5 (2.3)	7.7 (3)	0.083
BI 5 (bowel control)	9.2 (1.9)	9.6 (1.4)	0.083	9.1 (2)	9.2 (1.9)	0.66
BI 6 (bladder control)	9.2 (1.9)	7.8 (3)	**0.006**	8.6 (2.3)	7.5 (3.2)	**0.011**
BI 7 (toilet use)	9.7 (1.2)	9.7 (1.2)	1.0	8.5 (2.6)	8.3 (2.6)	0.71
BI 8 (transfers)	14.3 (1.8)	13.6 (2.6)	0.096	12.4 (3.7)	12 (4)	0.474
BI 9 (mobility on level surfaces)	14.3 (1.8)	13.3 (3.2)	0.109	11.8 (4)	10.2 (4.9)	**0.036**
BI 10 (stairs)	7.8 (3)	7.6 (3.3)	0.80	5.2 (3.5)	5.2 (3.5)	1.00
Functional Dependency			0.929			**0.029**
Severe	1 (3)	1 (3)		10 (20)	12 (24)	
Moderate	12 (30)	16 (41)		25 (49)	17 (33)	
Slight	8 (21)	9 (23)		11 (22)	9 (18)	
Functional Independence	18 (46)	10 (26)		5 (10)	4 (9)	
EQ-5D-5L ^b^ total score	7.4 (2.1)	8.8 (2.9)	**0.002**	11.1 (4.1)	12.7 (4.7)	**0.011**
Mobility	1.6 (0.9)	1.8 (1)	0.324	2.8 (1)	2.7 (1.1)	0.570
Self-care	1.1 (0.3)	1.4 (0.6)	**0.01**	1.9 (1.2)	2.2 (1.2)	**0.041**
Usual activities	1.3 (0.5)	1.7 (1)	**0.026**	2.5 (1.2)	2.8 (1.4)	0.140
Pain/Discomfort	1.8 (0.9)	2.1 (1)	0.086	2.2 (1)	2.7 (1.1)	**0.005**
Anxiety/Depression	1.5 (0.9)	1.8 (0.9)	0.079	1.8 (1.1)	2.3 (1.1)	**0.026**

Notes: Variables in bold are significant at *p* < 0.05. ^a^ Barthel Index; ^b^ European Quality of Life -5 dimensions-5 levels.

**Table 4:** Mean differences regarding functional status and QoL within middle-aged group at follow-up. Quantitative variables are expressed as mean (M) and standard deviation (SD), and qualitative variables as a percentage.

		Middle-Aged Group				
	Baseline Non-Frail (n = 61)	Follow-Up Non-Frail (n = 57)	*p*-Value	Baseline Frail (n = 28)	Follow-Up Frail (n = 24)	*p*-Value
	Mean (SD) or % (n)			Mean (SD) or % (n)		
DASI ^a^ total score	43 (13.4)	39 (15.4)	**0.008**	22.9 (17.3)	20.4 (17.1)	0.343
DASI 1 (self-care)	2.7 (0.4)	2.6 (0.6)	0.248	2.3 (1)	2.1 (1.1)	0.469
DASI 2 (walking indoors)	1.8 (0)	1.8 (0)	1.00	1.5 (0.6)	1.6 (0.5)	0.328
DASI 3 (walking on level ground)	2.8 (0)	2.6 (0.6)	0.083	2.2 (1.1)	2.1 (1.2)	0.328
DASI 4 (climbing stairs)	5.1 (1.4)	4.8 (1.8)	0.083	3.2 (2.8)	3 (2.8)	0.575
DASI 5 (running a short distance)	4.6 (4)	3.9 (4)	0.168	1.3 (3)	2 (3.6)	0.328
DASI 6 (doing light work around the house)	2.7 (0.4)	2.6 (0.5)	0.322	2.4 (0.9)	1.9 (1.3)	**0.043**
DASI 7 (doing moderate work around the house)	3.3 (0.9)	3.1 (1.1)	0.322	2.2 (1.7)	1.9 (1.8)	0.328
DASI 8 (doing heavy work around the house)	6.7 (3)	5.3 (3.8)	**0.016**	2 (3.5)	1 (2.7)	0.083
DASI 9 (doing garden work)	3.4 (2)	3.2 (2)	0.484	2.1 (2.3)	1.9 (2.3)	0.664
DASI 10 (sexual relations)	3.7 (2.4)	3.1 (2.6)	0.057	1.5 (2.4)	1.1 (2.2)	0.328
DASI 11 (participating in moderate recreational activities)	3.6 (3)	3.6 (3)	1.00	1 (2.3)	1.5 (2.7)	0.328
DASI 12 (participating in strenuous sports)	2.6 (3.6)	2.1 (3.4)	0.209	0.6 (2.2)	0.3 (1.5)	0.328
Functional capacity			0.601			0.070
Autonomy	15 (25)	9 (19)		2 (7)	(4)	
Good	30 (49)	26 (43)		3 (11)	5 (20)	
At risk	16 (26)	22 (36)		23 (82)	18 (64)	
EQ-5D-5L ^b^ total score	7.2 (2)	9 (3.3)	***p* < 0.001**	10.5 (4.2)	14.1 (5.2)	***p* < 0.001**
Mobility	1.3 (0.6)	1.7 (0.9)	***p* < 0.001**	2.3 (1.2)	2.9 (1.4)	**0.003**
Self-care	1.1 (0.3)	1.4 (0.8)	***p* < 0.001**	1.7 (1)	2.4 (1.3)	***p* < 0.001**
Usual activities	1.3 (0.7)	1.6 (0.9)	**0.020**	2.1 (1.2)	2.7 (1.2)	***p* < 0.001**
Pain/Discomfort	2 (0.9)	2.4 (1.1)	**0.007**	2.3 (0.9)	3.3 (0.8)	***p* < 0.001**
Anxiety/Depression	1.5 (0.8)	1.9 (1.1)	**0.015**	2.2 (1)	2.6 (1)	0.069

Notes: Variables in bold are significant at *p* < 0.05. ^a^ Duke Activity Status Index; ^b^ European Quality of Life-5 dimensions-5 levels.

**Table 5:** Results of predictors for frailty in elderly CVD group.

Variables ^1^	Sig.	Logistic Regression Analysis OR	95% C.I. for Exp (B)	
			Lower	Upper
BI 4	**0.119**	0.736	0.460	1.175
BI 7	0.717	1.103	0.651	1.868
BI 8	0.467	1.145	0.794	1.651
BI 9	0.992	0.998	0.705	1.414
BI 10	0.651	1.053	0.841	1.319
Mobility	**0.040**	2.344	1.038	5.293
Self-care	0.226	2.598	0.554	12.184
Usual activities	0.156	2.002	0.767	5.222
Age category	0.106			
Age category (1)	0.797	0.776	0.112	5.381
Age category (2)	**0.035**	7.655	1.150	50.969
Age category (3)	0.542	1.885	0.245	14.478
Age category (4)	0.246	5.505	0.308	98.506
Gender	0.070	0.260	0.061	1.115

^1^ Variable(s) entered on step 1: BI 4, BI 7, BI 8, BI 9, BI 10, Mobility, Self-care, Usual activities, Age category, Gender. Notes: Variables in bold are significant at *p* < 0.05.

**Table 6:** Results of predictors for frailty in the middle-aged CVD group.

Variables ^1^	Sig.	Logistic Regression Analysis OR	95% C.I. for Exp (B)	
			Lower	Upper
DASI 4	0.054	0.690	0.473	1.007
DASI 6	0.575	0.687	0.185	2.554
DASI 7	0.462	1.257	0.683	2.316
DASI 8	**0.002**	0.698	0.557	0.873
DASI 9	0.076	1.469	0.961	2.245
DASI 10	0.177	0.822	0.628	1.075
Mobility	**0.022**	2.576	1.148	5.783
Age category	0.221			
Age category (1)	0.219	7.44	0.303	182.34
Age category (2)	0.134	11.55	0.471	283.42
Age category (3)	**0.042**	64.18	1.17	3532.14
Gender	0.879	1.120	0.261	4.819

^1^ Variable(s) entered on step 1: DASI 4, DASI 6, DASI 7, DASI 8, DASI 9, DASI 10, Mobility, Age category, Gender. Notes: Variables in bold are significant at *p* < 0.05.

## Data Availability

The data presented in this study are available on request from the corresponding author. The data are not publicly available due to ethical restrictions.

## Supplementary Materials

The following are available online at https://www.mdpi.com/article/10.3390/ijerph19042286/s1, Table S1: Correlations between falls, functional status measured with Barthel Index (BI), and age category in elderly group. Table S2: Correlations between Barthel Index (BI) and Quality of life (QoL) domains in non-frail elderly subgroup at baseline and follow-up. Table S3: Correlations between cardiac valvulopathies, BI (total score), BI 6, BI 10, and QoL (total score) in CVD non-frail elderly patients. Table S4: Correlations between Barthel Index (BI) and Quality of life (QoL) domains in frail elderly subgroup at baseline and follow-up. Table S5: Correlations between falls, frailty, and functional capacity (measured with Duke Activity Status Index (DASI) in middle-aged group. Table S6: Correlations between ischemic heart disease, DASI (total score), DASI 5, DASI 7, DASI 11, DASI 12, and QoL (total score) in CVD non-frail middle-aged patients. Table S7: Correlations between Duke Activity Status Index (DASI) and Quality of life (QoL) domains in non-frail middle-aged subgroup at baseline and follow-up. Table S8: Correlations between heart failure, DASI 3 (walking on level ground) and QoL (total score) in CVD frail middle-aged patients. Table S9: Correlations between Duke Activity Status Index (DASI) and Quality of life (QoL) domains in frail middle-aged subgroup at baseline and follow-up.
