# Supplementary material for: Exploring the Relationship between Frailty, Functional Status, Polypharmacy, and Quality of Life in Elderly and Middle-Aged Patients with Cardiovascular Diseases: A One-Year Follow-Up Study
Source: Int J Environ Res Public Health. 2022 Feb 17;19(4):2286. doi: 10.3390/ijerph19042286 (PMC8871852; doi:10.3390/ijerph19042286)
Supplement: Supplementary file 1 [file ijerph-19-02286-s001.zip › ijerph-1562781-supplementary.pdf]

Table S1 Correlations between falls, functional status measured with Barthel Index (BI), and age category in elderly group

**Variables correlated with falls (baseline)**	**Correlation Coefficient (r)**	**Sig.** ***(P value)***
BI total score	-.448	***p<0.001***
BI 1 (feeding)	-.222	***.036***
BI 2 (bathing)	-.181	.087
BI 3 (grooming)	-.169	.111
BI 4 (dressing)	-.220	***.037***
BI 5 (bowel control)	-.129	.227
BI 6 (bladder control)	-.229	***.004***
BI 7 (toilet use)	-.256	***.015***
BI 8 (transfers)	-.363	***p<0.001***
BI 9 (mobility on level surfaces)	-.324	***.002***
BI 10 (stairs)	-.187	.077
Age category ≥ 65 - 69	-.281	.243
Age category 70 - 74	-.516	***.012***
Age category 75 - 79	-.356	.114
Age category 80 - 84	-.258	.300
Age category ≥ 85	-.551	.124
BI total score in non-frail elderly	-.162	.325
BI total score in frail elderly	-.476	***p<0.001***

Notes: **Variables in bold** are significant at $p < 0.05$.

Table S2 Correlations between Barthel Index (BI) and Quality of life (QoL) domains in non-frail elderly subgroup at baseline and follow-up

**Variables correlated with BI (baseline)**	**Correlation Coefficient (r)**	**Sig.** ***(P value)***
Mobility	-.288	.075
Self-care	-.477	***.002***
Usual activities	-.279	.085
Pain	-.267	.100
Anxiety/Depression	.101	.539
**Variables correlated with BI (follow-up)**	**Correlation Coefficient (r)**	**Sig.** ***(P value)***
Mobility	-.556	***p<0.001***
Self-care	-.497	***.002***

Usual activities	-.393	***.018***
Pain	-.391	***.018***
Anxiety/Depression	.046	.792

Notes: **Variables in bold** are significant at $p < 0.05$.

Table S3 Correlations between cardiac valvulopathies, BI (total score), BI 6, BI 10, and QoL (total score) in CVD non-frail elderly patients

**Variables correlated with cardiac valvulopathies (baseline)**	**Correlation Coefficient (r)**	**Sig.** ***(P value)***
BI (total score)	-.257	.115
BI 6 (bladder control)	-.350	***.029***
BI 10 (ascending and descending stairs)	-.481	***.002***
QoL (total score)	.159	.335

Notes: **Variables in bold** are significant at $p < 0.05$.

Table S4 Correlations between Barthel Index (BI) and Quality of life (QoL) domains in frail elderly subgroup at baseline and follow-up

**Variables correlated with BI (baseline)**	**Correlation Coefficient (r)**	**Sig.** ***(P value)***
Mobility	-.577	***p<0.001***
Self-care	-.698	***p<0.001***
Usual activities	-.529	***p<0.001***
Pain	-.268	.058
Anxiety/Depression	-.280	***.046***
**Variables correlated with BI (follow-up)**	**Correlation Coefficient (r)**	**Sig.** ***(P value)***
Mobility	-.736	***p<0.001***
Self-care	-.807	***p<0.001***
Usual activities	-.784	***p<0.001***
Pain	-.666	***p<0.001***
Anxiety/Depression	-.337	***.029***

Notes: **Variables in bold** are significant at $p < 0.05$.

Table S5 Correlations between falls, frailty, and functional capacity (measured with Duke Activity Status Index (DASI) in middle-aged group

**Variables correlated with falls (baseline)**	**Correlation Coefficient (r)**	**Sig.** ***(P value)***
DASI total score	-.316	***.003***
DASI 1 (self-care)	.058	.586
DASI 2 (walking indoors)	.100	.350
DASI 3 (walking on level ground)	-.067	.531
DASI 4 (climbing stairs)	-.003	.679
DASI 5 (running a short distance)	-.347	***.001***
DASI 6 (doing light work around the house)	.100	.350
DASI 7 (doing moderate work around the house)	-.063	.557
DASI 8 (doing heavy work around the house)	-.283	***.007***
DASI 9 (doing garden work)	-.244	***.021***
DASI 10 (sexual relations)	-.319	***.002***
DASI 11 (participating in moderate recreational activities)	-.375	***p<0.001***
DASI 12 (participating in strenuous sports)	-.201	***.059***
DASI total score in non-frail elderly	-.416	***.001***
DASI total score in frail elderly	-.038	.849

Notes: **Variables in bold** are significant at $p < 0.05$.

Table S6 Correlations between ischemic heart disease, DASI (total score), DASI 5, DASI 7, DASI 11, DASI 12, and QoL (total score) in CVD non-frail middle-aged patients

**Variables correlated with ischemic heart disease (baseline)**	**Correlation Coefficient (r)**	**Sig.** ***(P value)***
DASI (total score)	-.322	***.011***
DASI 5 (running a short distance)	-.330	***.009***
DASI 7 (doing moderate work around the house)	-.307	***.016***
DASI 11 (participating in moderate recreational activities)	-.293	***.022***
DASI 12 (participating in strenuous sports)	-.276	***.032***
QoL (total score)	.067	.607

Notes: **Variables in bold** are significant at $p < 0.05$.

Table S7 Correlations between Duke Activity Status Index (DASI) and Quality of life (QoL) domains in non-frail middle-aged subgroup at baseline and follow-up

**Variables correlated with DASI (baseline)**	**Correlation Coefficient (r)**	**Sig.** ***(P value)***
Mobility	-.149	.251
Self-care	-.152	.242
Usual activities	.004	.976
Pain	.101	.440
Anxiety/Depression	-.174	.180
**Variables correlated with DASI (follow-up)**	**Correlation Coefficient (r)**	**Sig.** ***(P value)***
Mobility	-.480	***p<0.001***
Self-care	-.412	***.001***
Usual activities	-.528	***p<0.001***
Pain	-.381	***.003***
Anxiety/Depression	-.148	.272

Notes: **Variables in bold** are significant at $p < 0.05$.

Table S8 Correlations between heart failure, DASI 3 (walking on level ground) and QoL (total score) in CVD frail middle-aged patients

**Variables correlated with heart failure (baseline)**	**Correlation Coefficient (r)**	**Sig.** ***(P value)***
DASI (total score)	.280	.149
DASI 3 (walking on level ground)	.521	***.004***
QoL (total score)	-.148	.454

Notes: **Variables in bold** are significant at $p < 0.05$.

Table S9 Correlations between Duke Activity Status Index (DASI) and Quality of life (QoL) domains in frail middle-aged subgroup at baseline and follow-up

**Variables correlated with DASI (baseline)**	**Correlation Coefficient (r)**	**Sig.** ***(P value)***
Mobility	-.629	***p<0.001***
Self-care	-.561	***.002***
Usual activities	-.503	***.006***
Pain	-.637	***p<0.001***
Anxiety/Depression	-.381	***.045***
**Variables correlated with DASI (follow-up)**		

Mobility	-.583	***.003***
Self-care	-.763	***p<0.001***
Usual activities	-.647	***.001***
Pain	-.667	***p<0.001***
Anxiety/Depression	-.544	***0.006***

Notes: **Variables in bold** are significant at $p < 0.05$.
